# Resolving selfish and spiteful interdependent conflict

**DOI:** 10.1098/rspb.2024.0295

**Published:** 2024-04-10

**Authors:** Alexander J. Stewart, Charlie Pilgrim, Nichola J. Raihani

**Affiliations:** ^1^ School of Mathematics and Statistics, University of St Andrews, St Andrews, UK; ^2^ Department of Experimental Psychology, University College London, 26 Bedford Way, London WC1H 0AP, UK; ^3^ School of Psychology, University of Auckland, 23 Symonds Street, Auckland, 1011, New Zealand

**Keywords:** conflict, interdependence, spite, cooperation, perception

## Abstract

Interdependence occurs when individuals have a stake in the success or failure of others, such that the outcomes experienced by one individual also generate costs or benefits for others. Discussion on this topic has typically focused on positive interdependence (where gains for one individual result in gains for another) and on the consequences for cooperation. However, interdependence can also be negative (where gains for one individual result in losses for another), which can spark conflict. In this article, we explain when negative interdependence is likely to arise and, crucially, the role played by (mis)perception in shaping an individual's understanding of their interdependent relationships. We argue that, owing to the difficulty in accurately perceiving interdependence with others, individuals might often be mistaken about the stake they hold in each other's outcomes, which can spark needless, resolvable forms of conflict. We then discuss when and how reducing misperceptions can help to resolve such conflicts. We argue that a key mechanism for resolving interdependent conflict, along with better sources of exogenous information, is to reduce reliance on heuristics such as stereotypes when assessing the nature of our interdependent relationships.

## 1. Introduction

Humans spend their lives trying to navigate a tangled web of cause and effect. This has rarely been more evident than today, in the era of the polycrisis [1], when seemingly disparate challenges combine to disrupt the lives of individuals and institutions across the globe. Successfully navigating these problems requires investment in public goods at scales that cut across communities and cultural boundaries. This is only possible if there is a perception of shared prosperity—a rising tide lifts all boats—rather than a competitive, beggar-thy-neighbour view of the world.

Interdependence is an umbrella term which captures the idea that benefits or harms to others may benefit or harm you in turn [2–7]. The concept of interdependence is not limited to humans, and has frequently been explored in the context of biological evolution (e.g. [4,5,8]), sharing commonalities with concepts such as indirect genetic effects (where gene expression in one organism can influence the expression of traits in another [9]). Indeed, any agents that interact repeatedly are likely to be interdependent to some degree [8], since the outcome of subsequent interactions may depend on what happened previously [10–15]. However, in the complex social interactions of humans, occurring across disparate domains and timescales, it is often the overall *perception* of interdependence between one individual or group and another that determines the extent of observed cooperation or conflict [6,16–20].

Interdependence can be perceived as positive (where an individual expects that another's benefit will benefit them) or negative (where an individual expects that another's benefit will harm them). When interdependence is perceived as positive, we expect to observe more synergistic, cooperative behaviours, and when it is perceived as negative, we expect to observe more competitive, hostile behaviours (hereafter ‘conflict’). This review focuses on how humans perceive their interdependence with others, when those perceptions turn into *mis*perceptions that generate unnecessary conflict, and how such misperceptions can be resolved.

One difficulty in assessing the nature and consequences of interdependence is that the knock-on effects of one's actions can differ across contexts and timescales [21]. From an evolutionary perspective, fitness interdependence can be defined as ‘the degree to which two or more organisms influence each other's success in replicating their genes’ [5, p. 429]. The evolutionary consequences of interdependence therefore occur on a generational timescale [11,14]. However, shorter-term consequences of interdependence may also affect decision-making, and can be especially relevant for understanding cooperation and conflict in humans. Short-term gains or losses from helping or harming others (e.g. monetary benefits/costs) can affect an individual's objective wealth and resources, their subjective well-being, or both. We refer to this overall state of an individual—integrating objective resources and subjective well-being—as their utility (box 1) [23]. As with fitness interdependence, helping or harming another may have interdependent effects on one's utility (e.g. by encouraging future acts of reciprocity [24–26]). And so utility interdependence is simply the degree to which an individual's utility is impacted by the outcomes experienced by, or actions of, others (box 1). Whereas fitness interdependence is relevant to the action of natural selection across generations, utility interdependence is relevant to an individual's decision-making within their lifetime, shaped by processes such as learning or rational decision-making [27–31].

Box 1. **Types of interaction arising due to interdependence.**

Interdependence can result in qualitatively different types of interaction depending on the payoffs associated with a focal individual's action, *w*_a_, the payoffs the recipient of the action receives as a result, *w*_r_, and the stake, *s*, the focal actor holds in the recipient. The conditions for these different types of action to be favoured (either by selection or for a rational actor) are summarized in table 1. These conditions are analogous to the conditions favouring different types of action between related individuals [22] (where the payoffs associated with actions may refer to an individual's lifetime fitness, or alternatively may refer to the product of a rational decision process or a learning process aimed at maximizing utility) and the expressions are therefore analogies of Hamilton's rule. The different types of actions that arise owing to interdependence can be described qualitatively as follows:

**Interdependent altruism:** when an actor pays a cost, *w*_a_ < 0, to produce a benefit to a recipient, *w*_r_ > 0. This type of action can only be favoured if the actor has a sufficiently large, positive stake in the recipient. If this condition is met, the downstream consequences of providing a benefit to the recipient are beneficial for the original actor to an extent that outweighs the original cost.

**Interdependent mutualism:** when an actor does something that provides them with a direct benefit, *w*_a_ > 0, which also produces a benefit to a recipient, *w*_r_ > 0. Such actions are always favoured when the stake is positive, since both the downstream consequences of the original action, and the original action itself provide benefits to the focal actor. However, such actions can also be favoured when the stake between two individuals is negative. In this scenario, the downstream consequences of the original action are costly to the original actor, but those costs are outweighed by the initial benefits of the action.

**Interdependent selfishness:** when an actor does something that provides them with a direct benefit, *w*_a_ > 0, but generates a cost for the recipient, *w*_r_ < 0. Such actions are always favoured when the stake is negative, since the downstream consequences of the original action are beneficial for the original actor, as is the original action itself. However, such actions can also be favoured when the stake between two individuals is positive. In this scenario, the downstream consequences of the original action are also costly to the original actor, but those costs are outweighed by the initial benefits of the action.

**Interdependent spite:** when an actor pays a cost, *w*_a_ < 0, to produce a cost for a recipient, *w*_r_ < 0. This type of action can only be favoured if the actor has a sufficiently large, negative stake in the recipient. If this condition is met, the downstream consequences of inflicting a cost on the recipient are ultimately beneficial for the original actor to an extent that outweighs the original cost.

Of course, utility and fitness interdependence may often align. For example, in war, an individual might be positively interdependent with members of their own battalion (and negatively interdependent with enemy battalions) owing to their immediate interest in survival, their ideological preferences, and their desire to protect their family. However, the different forms of interdependence may also misalign, for example around action on climate change, where short-term profit-seeking can erode the long-term quality of the environment, jeopardizing our future health and survival. The context-specific nature of interdependence highlights the importance of individual perceptions, since a shift in focus from short-term utility to long-term utility or to fitness interdependence may result in radically different behaviours and can help us to understand how and when conflict will arise in the first place [32].

The concept of interdependence then is shorthand for the combined effects of what are, especially in humans, highly complex and nuanced social interactions playing out across time, space and context. While this level of generality can make the exact nature of interdependence hard to pin down, we argue that this is precisely the challenge humans face when making decisions about how to interact with one another: humans often make assessments based on perceptions of interdependence without knowing the full ramifications of their actions. Perceptions can be imperfect, and where misperception leads to a belief that interdependence is negative, this can generate conflict [21,33,34]. We discuss how resolving such conflicts requires either an exogenous source of better information, or the evolution of improved perceptions.

## 2. Positive interdependence and relatedness

Helping behaviour can be favoured (and is most likely to arise) when individuals are sufficiently positively interdependent with interaction partners. The conditions favouring helping behaviour are best understood by analogy with Hamilton's rule [35,36], which describes when natural selection will favour the evolution of helping behaviour as *rB* > C, where *C* is the personal fitness cost paid by the helper, *B* is the fitness benefit to recipients, and *r* is the genetic relatedness between helper and recipients. Relatedness is the chance of a given allele being shared by interaction partners over and above the chance that any two individuals drawn at random from the population will share that allele [22,37,38]. Hamilton's rule captures the idea that altruistic strategies can be favoured, despite imposing direct reproductive costs on altruists, so long as such costs are sufficiently outweighed by fitness benefits to relatives [37,38]. Classic examples of altruism evolving via kin selection include worker sterility in eusocial insects, and helpers in cooperative breeders delaying reproduction and investing in helping raise relatives [39].

The decision of whether to engage in altruistic behaviour also depends on the perceived or estimated costs and benefits. For example, *Megaponera* ants, when injured during raids on termite colonies, do not receive help from their colony mates if they are either too old or too badly injured [40]. Despite being equally related to potential recipients, the net benefit of helping a badly injured or aged ant is insufficient to favour the evolution of altruism. Similarly, long-tailed tits help to raise young at relatives' nests, but only if their own breeding attempts fail (i.e. when the cost of helping in terms of lost personal reproduction is minimal [41]). However, in many contexts, estimating how downstream effects will filter back to the focal individual or their kin may be quite uncertain, especially if those effects are temporally delayed [3,7], whereas the direct cost of helping or harming another may be clear.

**Figure 1.**
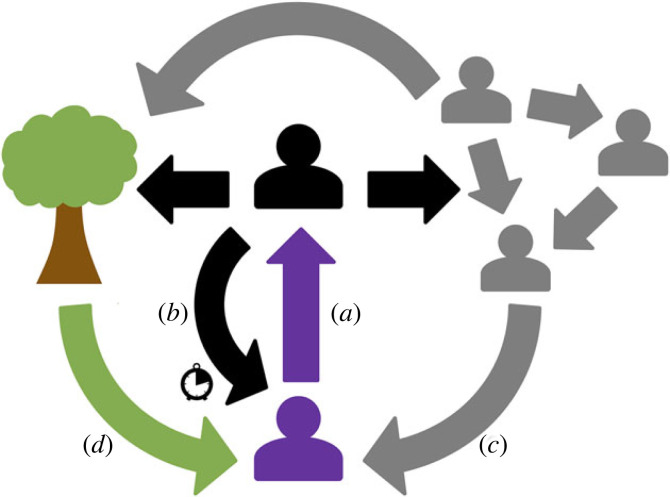
Downstream effects: the stake, *s*, a focal individual (purple) holds in another individual (black), depends on how the actions of the purple individual towards the black individual eventually feed back to affect them. (*a*) The focal individual's initial action (purple arrow) may help or harm the target. The downstream consequences of this may manifest via (*b*) direct actions by the recipient towards the focal actor later in time (black arrow) (eg. direct reciprocity). Downstream consequences of an initial action might also affect how recipients behave with others (grey individuals) (*c*), with outcomes that feed back indirectly to the focal individual (e.g. network effects or indirect reciprocity [42]). (*d*) Downstream effects may also produce an impact via knock-on effects through the shared environment of the actors that do not involve direct actions by black or grey individuals toward the purple actor.

Analogies with Hamilton's rule are helpful for understanding how interdependence might shape cooperation and conflict. Instead of considering just the relatedness between individuals, we can consider the overall degree to which they influence one another's outcomes, including through delayed, indirect and environmentally mediated effects (figure 1). Although there are myriad ways that the effects of interdependence can manifest, ultimately it is the overall effect that matters. This overall effect can be described as the ‘stake’, *s*, an individual has in their social partners [4]. The concepts of stake and relatedness are superficially similar. Relatedness describes the genetic stake an individual has in the reproductive success of a social partner, with interdependence being a more generalized version of the stake an individual has in a social partner, via either shared genes or shared fate [43]. Relatedness and stake are both relative quantities and both can be either positive or negative. By analogy with Hamilton's rule, we can state that the tendency for a focal individual to pay a cost *C* to generate a benefit *B* for a recipient will be under positive selection if *sB* > *C* [4] (box 1).

Nonetheless, the real-world manifestations of the effects of kin selection and of interdependence are typically quite different. When considering kin selection, we are explicitly concerned with fitness effects, whereas we can also think about the effects of interdependence in terms of utility—i.e. in a non-evolutionary setting. This has implications for the ways that individuals benefit via their partners' success: individuals who have a genetic stake in a partner benefit via the partner's reproductive benefits; whereas interdependent individuals benefit via the partner being able to continue behaving in ways that benefit the individual. In other words, the benefits of investing in interdependent partners may be contingent upon the partner's survival or short-term state and not their reproductive success or fitness, *per se*.

Relatedness and stake also differ in some important ways. Relatedness, while often difficult to quantify in practice [44,45], is an objective measure that is fixed in time. By contrast, stake combines several disparate downstream effects of an action, which may vary in time and across contexts ([8], box 2), e.g. benefits that accrue owing to reciprocity [25], the future benefits of shared public goods [32], and environmental robustness [46]. Unlike relatedness, the stake an individual holds in another's success may be positive in one context and negative in another. For example, two football players who belong to the same club, but different international teams, are positively interdependent when they play on the same side, and negatively interdependent when they play in opposition. Because interdependence is a complex and multifaceted concept that changes in response to environmental conditions and shifting norms of behaviour, it is heavily influenced by the perceptions and past experience of those making decisions [25]. This can have important consequences for perceptions of interdependence as simple heuristics for detecting and recognizing kin (e.g. being raised in the same social group/family) may not suffice when it comes to tracking how one's *stake* in social partners fluctuates over time or across contexts. Keeping track of fine-grained differences in the degree of interdependence with another individual may also be impractical, or cognitively too costly. Whether individuals make assessments based on an overall average, or based on the specific context of an interaction, may itself be seen as a strategic decision, or else as a trait able to evolve under natural selection (e.g. [47]).

Box 2. **Using individual assessment to reduce conflict.**

**Figure 2.**
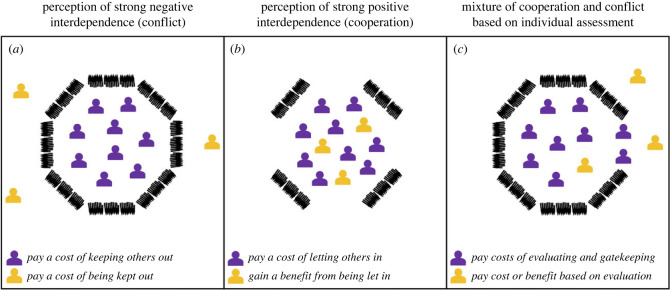
Perceptions of interdependence. (*a*) When members of the purple group perceive strong negative interdependence with members of the yellow out-group, they are willing to engage in spiteful interdependent conflict, and pay a cost to inflict a cost on the out-group (table 1). In this example, the purple group believes they are better off if they pay a cost to keep yellow group members outside of their community, resulting in a cost to the out-group members. (*b*) When the purple group perceives strong positive interdependence with the yellow out-group, they are willing to engage in altruistic interdependent cooperation, and pay a cost to generate a benefit for the out-group. In this example the purple group believes they are better off if they pay the cost of welcoming all out-group members into their community, generating a benefit to the out-group members. (*c*) When the purple group evaluates out-group members one-by-one to determine their individual degree of interdependence, they must pay the cost of evaluating, alongside the costs associated with individual positive or negative interdependent relationships. In this example the purple group must first evaluate the out-group members as they try to enter, and then pay the costs of gatekeeping i.e. keeping out those perceived as negatively interdependent and letting in those perceived as positively interdependent. Whether evaluation is worthwhile depends on the size of the cost of evaluation, compared with the costs of incorrectly perceiving some out-group members when no evaluation takes place.

A major source of misperception when trying to determine the degree of interdependence with another individual is reliance on heuristics or group-level information, rather than individual-level information, for example using group-level stereotypes to assess an individual's trustworthiness. While the use of heuristics inevitably leads to inaccurate individual-level perceptions of interdependence, performing an individual-level assessment can be costly. Figure 2 illustrates the role of individual- versus group-level assessments in shaping the perceptions and decisions of a focal individual. Here we explore the efficacy of individual-level assessment as a tool for reducing conflict, using a simple model.

**Group-level assessment:** We first assume that a focal individual must decide how to interact with a target individual. The focal individual has some information about the target, based on their group identity. In particular, the focal individual knows that the target individual's group contains some individuals with whom they are negatively interdependent, such that the stake they hold in these individuals is *s*_b_ < 0. However, the remaining individuals in the group are positively interdependent with the focal individual, such that the stake they hold in such individuals is *s*_g_ > 0. And so, the expected utility associated with an action by the focal individual towards a randomly selected member of the group, *U*_a_, is given by

2.1 $$\left\langle U_{a}\right\rangle =w_{a}+(s_{g}p+s_{b}(1-p))w_{r,}$$

where *p* is the perceived proportion of positively interdependent individuals in the group, *w*_a_ is the payoff associated with a focal individual's action and *w*_r_ is the payoff the recipient of the action receives as a result (box 1). If the focal individual only makes decisions based on group-level information, they will engage in interdependent spite with *all* group members if

2.2 $$\left|\frac{w_{a}}{w_{r}}\right|<-(s_{g}p+s_{b}(1-p)).$$

Similarly, they will engage in interdependent altruism with *all* group members if

2.3 $$\left|\frac{w_{a}}{w_{r}}\right|<(s_{g}p+s_{b}(1-p)).$$

Similar conditions can easily be written down for interdependent selfishness and mutualism, but here we will focus on spiteful and altruistic interactions.

**Individual-level assessment:** Next we consider individual-level assessment, under the assumption that the true proportion of positively interdependent individuals in the group is *q*, and the focal individual, when performing an individual-level assessment, is accurate with probability *ρ*. The expected utility associated with an action by the focal individual towards a randomly selected member of the group when using individual assessment, *U*_A_, is given by

2.4 $$\left\langle U_{A}\right\rangle =(q+\rho -2q\rho )w_{a}+(q(1-\rho )s_{g}+(1-q)\rho s_{b})w_{r}.$$

If the focal individual makes decisions based on individual-level information, they will engage in interdependent spite with those who are perceived as negatively interdependent (but not with those who are perceived as positively interdependent) if similarly, they will

2.5 $$\left|\frac{w_{a}}{w_{r}}\right|<-\frac{q(1-\rho )s_{g}+(1-q)\rho s_{b}}{q+\rho -2q\rho }.$$

Similarly, they will engage in interdependent altruism with those who are perceived as positively interdependent (but not with those who are perceived as negatively interdependent) if

2.6 $$\left|\frac{w_{a}}{w_{r}}\right|<(s_{g}p+s_{b}(1-p)).$$

**The efficacy of individual assessment for resolving conflict:** If there is no individual-level assessment, and equation (2.2) is satisfied, then spiteful interdependent conflict will occur between the focal individual and group members. Since the group contains members who are in fact positively interdependent with the focal individual, some of this conflict is due to misperception. From equation (2.2), a necessary condition for conflict without individual assessment is

2.7 $$\left|\frac{s_{g}}{s_{b}}\right|<\frac{1-p}{p}.$$

From equation (2.5), conflict with those who are perceived to be negatively interdependent via individual assessment will never be advantageous if

2.8 $$\left|\frac{s_{g}}{s_{b}}\right|>\frac{\rho (1-q)}{q(1-\rho )}.$$

If we now define the *misperception of negative interdependence* by the focal individual as $\sigma_{n}=q-p$, we can combine equations (2.7) and (2.8) to derive a condition for individual assessment to assuredly remove spiteful conflict entirely as

2.9 $$\sigma_{n}>\frac{(1-q)q}{\rho /(2\rho -1)-q},$$

i.e. once misperceptions become big enough, individual assessment can resolve conflict entirely. A similar argument allows us to derive a condition for individual assessment to enable interdependent altruism when it is initially absent owing to misperceptions as

2.10 $$\sigma_{n}>\frac{(1-q)q}{\rho /(1-2\rho )-q}$$

(figure 3).

## 3. Negative interdependence as a source of conflict

If positive interdependence can generate the conditions for helping behaviours to evolve, negative interdependence can spark ‘manifest’ (observable) conflict [21]. Negative interdependence, *s* < 0, implies that the focal player hurts themself by helping another, over and above the direct cost of helping. The analogous scenario under kin selection, *r* < 0, can favour the evolution of ‘spite’, in which individuals pay a direct fitness cost to levy a greater fitness cost on a target, thereby indirectly benefitting relatives [22,48,49] (box 1).

Sufficiently negative interdependence can also lead to individuals harming others to produce downstream effects that benefit them or their group (box 1). We define *spiteful interdependent conflict* as occurring when an actor is willing to pay a cost, *w*_a_ < 0, to harm a recipient, *w*_r_ < 0. Implicitly, this means that the focal individual believes that they are negatively interdependent with the recipient of harm, such that the interdependent consequences of causing harm ultimately rebounds to their benefit (box 1). While the conditions that favour the evolution of spite may be relatively unusual [22,49,50], perceptions of strong negative interdependence leading to conflict are common among humans [17,51–55]. Crucially, because the stake one individual has in another describes the consequences of downstream effects (figure 1), which may not be fixed in time or are otherwise hard to predict, the perception an individual holds of their stake in another will often be wrong. The way actions impact upon focal individuals and their partners can also result in *selfish interdependent conflict*, where an individual pursuing their own interests (*w*_a_ > 0) generates costs for a partner (*w*_r_ < 0). Such selfish behaviour can arise, even among positively interdependent interaction partners, so long as interdependence is not too positive (box 1).

## 4. When does interdependence arise?

The stake that one individual has in another depends on a gamut of social and ecological factors, which we summarize briefly here (these are discussed in detail elsewhere [5,6,8,19,43]). Positive interdependence is most likely to arise among individuals who interact repeatedly with social partners, with the expected duration of the interaction resulting in a stronger stake in the partner. The availability of alternative interaction partners also influences how partners behave towards one another: the interdependent relationship with a social partner becomes more likely to favour the maintenance of helping behaviour as the cost of finding a replacement increases (box 1) [8,56]. Replacement costs are higher either when alternative partners are scarce, or when individuals ‘raise the stakes’ in cooperative interactions [57], such that more enduring relationships yield higher potential benefits than those that have just begun [25].

Positive interdependence also exists where individuals derive benefits from assorting with others. For example, group members can become interdependent because increased group size reduces predation risk (favouring cooperative investments to increase the size of one's social group, [58]) or because life in groups can buffer against the environmental extremes individuals might otherwise experience [59–61]. In some cases, this buffering is achieved via food sharing (e.g. [62,63]). Here, one's stake in social partners can increase as food availability becomes more unpredictable and decrease as individuals become more self-sufficient [63].

Resource scarcity can also generate negative interdependence, especially when competition is relatively local. Under local competition, one's immediate social partners are competing for the same scarce resources and any gains made by a neighbour translate directly into losses for the self [22]. This is analogous to the cancellation effect, which arises when local competition means that helping some relatives harms other relatives [64]. The extent of negative interdependence therefore depends on resource availability and on the way that resources are distributed in space. One example of how resource scarcity can spark conflict comes from behavioural games with pastoralists: those who are exposed to persistent resource scarcity (compared with those living in higher-yield areas) are twice as likely to behave antisocially in a joy-of-destruction game, by destroying a partner's earnings at no benefit to themselves [65]. Similarly, the extent of antisocial punishment (whereby players invest to punish cooperative interaction partners) is also more prevalent in countries with lower GDP [66], and other work has shown that civil conflict increases with resource scarcity [67,68].

Interdependence, by definition, depends on the extent to which interaction partners have a shared fate [69,70]. In extreme cases, where an individual's personal success is wholly dependent upon the success of the group, interdependence between members of the group will be highly positive [71,72]. But such a shared fate can often sharpen between-group competition, and therefore increase negative interdependence between members of competing groups [73,74].

## 5. The role of perception in generating interdependent conflict

Interdependent conflict arises under resource scarcity, when negative interdependence is sufficiently strong that one individual will pay a cost to directly harm another. The spatial and temporal distribution of resources is not generally easy to know, even if only a single type of resource such as food is at stake. Humans compete for a vast array of resources of different types, and the utility of a resource of one type typically depends on the availability of resources of another type. A clear example of temporal shifts in the perceived value of resources can be seen in the growing geopolitical competition over rare earth minerals as governments anticipate a shift away from fossil fuels towards green energy [75]. These shifts in perceptions are both a response to empirical evidence on global heating, and the product of a political and social discourse on how to respond to the crisis. In such scenarios, which involve anticipating the future political, economic, technological and physical state of the world, *perceptions* of interdependence are not a peripheral consideration, but the central source of conflict [34].

Perceptions of interdependence manifest through the need to estimate the stake, *s*, one individual holds in another, as well as the perceived consequences of an action for the actor and their target (estimates of *w*_a_ and *w*_r_, box 1). Much recent work has focused on perceptions of outcomes as described by interdependence theory [2,3,7]. Specifically, people perceive dyadic situations along dimensions of mutual dependence (the degree to which actions affect each other's outcomes), conflict (the degree to which preferred outcomes are in conflict) and power (which partner has the most influence over the outcome) [6,16,19,76,77]. These dimensions can be mapped from 2 × 2 payoff matrices, from which *w*_a_ and *w*_r_ can be estimated if we also have an estimate for our partner's expected behaviour.

Beyond immediate outcomes, people also perceive the degree of future interdependence and the amount of uncertainty in situations [16]. Uncertainty can apply to all aspects of a situation, including an interaction partner's preferences and the causal attribution of outcomes to a partner's intended actions (e.g. noise or mistakes in actions) [3,6]. In uncertain situations, monitoring partners' behaviour and associated payoffs from social interactions provides some cues as to interdependence [6,7]. Crucially, both the perception and reality of interdependence between one individual and an interaction partner can depend on the partner's perceptions. For example, in the context of a repeated game, in which two players repeatedly decide how to behave towards one another based on experience, ‘errors’ in the actions of one individual can lead to a perception of negative interdependence. Here, an initial misperception based on an error can generate an expectation about the future actions of an interaction partner, which leads ultimately to a correct perception of negative interdependence [78–80]. In scenarios like this, perceptions of interdependence are the result of a process of continual monitoring and updating [81]. A simple example of this kind of self-fulfilling prophesy arises between two players who use Tit-For-Tat in the infinitely iterated Prisoner's Dilemma. The dynamics of the game have three (quasi-) equilibrium states: both players defect, both players cooperate or both players alternate between cooperating and defecting. If both players (mistakenly) expect the other to defect in the first round, this becomes their fate for many rounds after. More generally, since repeated games contain many possible equilibrium states, the equilibrium players arrive at will inevitably be shaped by their perceptions of interdependence, which may in turn be shaped by errors.

However, in some cases we hypothesize that perceptions of interdependence are not based on the monitoring of another's actions, but instead on the identity (or social role [76]) of the interaction partner. This can include perceptions of genetic relatedness [43,82], or cultural norms, which determine the relationships and moral obligations people have to others. For example, many societies use different kin terms to describe family members [83] even though, in terms of relatedness, the relationships being described are equivalent [84]. Rather than describing relatedness or family ties, these culturally evolved terms might instead describe fitness interdependence between social partners [84]. The use of kin terms to label non-kin members of a social group (so-called ‘fictive kin’) might similarly signal interdependence [5]. Elsewhere, people use specific terms to denote interdependent interaction partners. For example, *osotua* relationships among Maasai herders are made with a small subset of the population and are used to label close relationships that are characterized by need-based sharing and, consequently, a high degree of interdependence [85,86]. *Hxaro* partnerships in Ju/’hoansi societies also serve a risk-pooling function [87] and the term may also signal positive interdependence among interaction partners [88].

When using identity heuristics to assess interdependence, it is not just perceptions about the availability of resources that drives conflict, but perceptions about the distribution of interdependent relationships we share with others (box 2). When we use a heuristic such as group identity to infer interdependence with others, we risk misperceiving the individual relationship, even if our perceptions of the overall group are accurate. Generating individual-level estimates of interdependence by monitoring individual actions may be preferable to using identity heuristics, since it reduces needless conflict of this type, but generating such estimates is likely to entail costs, in terms of time and cognition [47]. Where individual evaluation is costly, heuristics such as stereotypes become a double source of unnecessary interdependent conflict, since perceptions of stereotype groups tend to become more negative as the size of the group being stereotyped increases [47]. Coarser stereotypes (which are applied to larger numbers of people) are more likely to entail perceptions of negative interdependence *and* result in conflict with a greater number of people. We suggest that an important area of future research will focus on the role of factors such as information flow and cognitive load in shaping perceptions of interdependence, and in particular the ways that we can manage our information ecosystems to help shift assessments away from group-level judgements and towards the individual level.

Individual-level assessments, while preferable if accurate, are themselves subject to perception error via several cognitive mechanisms. For instance, individuals may misperceive a causal connection between negative payoffs for the self and positive payoffs for another when no causal relationship exists. Relatedly, a pervasive psychological tendency called ‘win–win denial’ occurs when individuals perceive positive payoffs for others as being bad for them, even when this is not the case [54]. For example, advantaged groups may perceive interventions to increase equality as necessarily harming them (e.g. [89]), even when the opposite is true [51]. Such misperceptions can result in a tendency to vote against equality-enhancing policies that would benefit the individuals who distrust them [90], to support policies or actions that enforce the *status quo* [91] or even harm the previously disadvantaged groups [89].

Regardless of whether identity heuristics or monitoring of individual actions are used, perceptions of interdependence are shaped by knowledge of the underlying environment, such as the distribution and future utility of resources. For example, a tendency to believe that benefits to self (or one's group) can only be obtained at the expense of another's misfortune (sometimes referred to as ‘zero-sum thinking’, [17,53]) is higher among individuals who experience resource scarcity, or who have experienced personal relative deprivation [92]. Economic stress can fuel perceptions of negative interdependence, reducing willingness to help others [93], increasing unethical behaviour [94], and fuelling affective polarization [95,96]. Misperceptions about temporal dynamics, such as failure to consider the longer-term outcomes of social interactions with others, can occur when individuals experience short-term costs in interactions with others and fail to perceive how positive outcomes for a partner over the longer term benefit them. Consequently, they mistakenly perceive negative interdependence with others, sparking conflict, when in fact they are positively interdependent with these partners in the long run. This type of temporal misperception is especially marked in the historical failure to take meaningful political action on climate change.

To give one example of how shifting perceptions can spark needless conflict, imagine a motorist who leaves their engine idling while parked. The motorist benefits from waiting in a climate-controlled environment (i.e. *w*_a_ > 0) but, in doing so, generates wider costs to others in terms of air pollution and greenhouse gas emissions (*w*_r_ < 0)—a classic ‘tragedy of the commons' scenario [97]. In this scenario of selfish interdependent conflict, benefits received by the motorist result in costs to others, but these benefits are not contingent on others experiencing these costs (i.e. the motorist would still benefit even if no one suffered). As such, the motorist is unlikely to engage in *spiteful* conflict by incurring a personal cost to further harm those outside. Nevertheless, spiteful interdependent conflict could emerge from this type of scenario. Individuals who experience costs due to the selfish actions of others might be incentivized to become ‘protesters’ and harm the selfish individuals, for example by incurring a cost to punish motorists who idle their engines. In this case, because *w*_a_ < 0 and *w*_r_ < 0, spiteful interdependent conflict emerges. This type of negative feedback can go still further if it changes the perceptions of interdependence within the scenario for the motorists, who now come to perceive negative interdependence with the protesters and consequently start to invest in actions specifically designed to harm them. Indeed, the (outlawed) practice of paying several thousand dollars to modify a vehicle diesel engine such that it emits more air pollution than it otherwise would (known as ‘rolling coal’) constitutes a real-world example of how such spiteful interdependent conflict can escalate and become based on identity heuristics rather than individual-level assessment.

In general, underlying uncertainty about the environment leads to uncertainty about the consequences of our actions. If we misperceive the costs of our actions to ourselves, or negative consequences of our actions for others (*w*_a_ and *w*_r_ in box 1), we may be more willing to engage in an action, even if we correctly assess the stake, *s*, we have in one another. Uncertainty of this type is at its greatest when we try to envisage how our actions might affect people we never meet, including future generations, and where consequences of actions play out in complex ways through e.g. supply chains, finance networks and environmental systems.

## 6. Resolving conflict

As we have emphasized throughout, interdependence, and the extent to which someone has a ‘stake’ in another's success, can be difficult to measure. Nonetheless interdependence is a useful framework for thinking about human decision-making because this reflects the real complexity and uncertainty of estimating the downstream consequences of our actions due to interdependence. This is especially true in everyday life, where we rarely have the time or the resources to make detailed assessments about the consequences of a social decision, and instead must rely on past experiences, intuitions and heuristics. The *perception* of interdependence is therefore central to understanding the causes of, and resolution to, the types of selfish and spiteful interdependent conflicts we have discussed so far. Of course, reducing uncertainty assists decision-making in general, and this is true for interdependence. More accurate perceptions of interdependence can be achieved through a better understanding of how actions relate to outcomes, discerning the intentions of interaction partners, recognizing the long-term and downstream effects of actions, and considering the broader context. Additionally, understanding the dynamics of interdependence (and how it can change) can aid in counterfactual and prospective thinking that can help individuals to make choices that avoid or resolve conflicts.

The idea that shifting perceptions can resolve conflict is, like the concept of interdependence itself, appealingly simple but sometimes hard to pin down. Where spiteful interdependent conflict is present, it is always sufficient to shift perceptions of the stake we have in someone else from negative to positive, and even if that is not possible, spiteful conflict can be resolved by reducing the strength of perceived negative interdependence (box 1). Selfish interdependent conflict may be harder to resolve, since we must convince people to forgo a benefit that comes at another's expense with the argument that the downstream consequences will outweigh the immediate benefits in the long run. Where conflict is the direct result of *mis*perceptions, we can hope to resolve things by providing access to more accurate information about the world [55]. However, where conflict is the result of accurate perceptions about the nature of interdependence, we must consider whether encouraging false perceptions of positive interdependence is justified, or whether such deception will backfire when the downstream consequences of foregoing conflict become clear. In the case of spiteful interdependent conflict that is not the result of misperception, alternative actions, such as negotiation, discussion, signalling intentions, improving the environment (for example by increasing resource availability), or bringing to bear external authorities such as laws or norms to curb negative interactions, might reduce the incentives to engage in harmful actions toward others.

**Figure 3.**
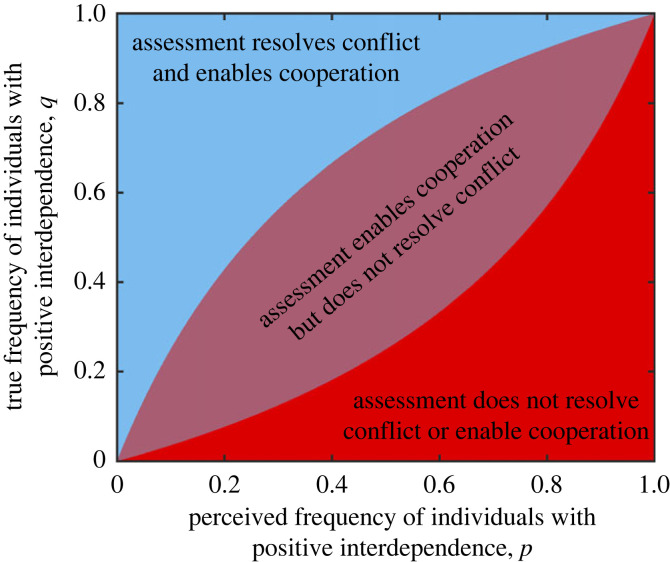
Resolving spiteful interdependent conflict through individual-level assessment. The figure shows the extent to which individual-level assessment can resolve spiteful interdependent conflict or promote interdependent altruism as a function of the frequency of perceived positively interdependent individuals in a group (*p*) and the actual frequency of positively interdependent individuals in the group (*q*) (box 2). In the blue region, individual-level assessment can both resolve spiteful conflict (equation (2.9)) and enable interdependent altruism (equation (2.10)). And so a focal individual engaging in individual-level assessment can be expected to cooperate with target group members they assess as positively interdependent, but will not engage in any spiteful conflict. In the purple region, individual-level assessment cannot always resolve spiteful conflict but can enable interdependent altruism. And so a focal individual engaging in individual-level assessment can be expected to cooperate with target group members they assess as positively interdependent, and to engage in spiteful conflict with those they assess as negatively interdependent. In the red region, individual-level assessment cannot always resolve spiteful conflict and cannot enable interdependent altruism. And so a focal individual engaging in individual-level assessment will not engage in any cooperation, but can be expected to engage in spiteful conflict with those they assess as negatively interdependent. The figure shows conditions for conflict and cooperation from equations (2.9) and (2.10), with a fixed accuracy of individual assessment ρ=0.75.

When considering ways to resolve interdependent conflict, the role of identity heuristics (e.g. stereotypes) in assessments of interdependence are of particular interest. Individual-based (rather than group-based) assessment of interdependence can reduce conflict by limiting selfish or spiteful interactions to only that subset of a group with whom actors are genuinely negatively interdependent, rather than maintaining conflict with all members of a group indiscriminately. In figure 2 and box 2 we construct a simple model of individual and group-based assessment, showing that individual-based assessment can limit spiteful interdependent conflict by shifting it away from the whole group to a subset. Moreover, under some circumstances, this can lead to the cessation of spiteful interdependent conflict altogether (box 2; figure 3). The ability of individual assessment to remove conflict depends on the accuracy of assessment as well as the level of misperception of the group *prior* to implementing individual assessment. For example, where perceptions of negative interdependence with a whole group arise because of actions by a small minority, this can lead to over-estimates of the numbers of negatively interdependent group members and group-level conflict, which exposes the vast majority to costs they would not incur if they were treated as individuals. Introducing individual assessment is guaranteed to resolve conflict entirely provided the initial misperception is sufficiently large (figure 3). A similar argument holds when altruism is withheld based on misperceptions of group-level interdependence (box 2).

**Table 1.** Types of interaction arising owing to interdependence. Different perceptions of interdependence lead to different types of social interactions emerging through lifetime fitness costs, rational decision-making or learning. Here we call the effect of an action on an instigator *w*_a_ and the effect on the action's recipient *w*_r_, with the qualitatively different types of interdependent relationships defined by analogy with Hamilton's rule. Interdependent mutualism, in which an action benefits both instigator and recipient, will emerge provided the perceived stake is not too negative, whereas interdependent altruism, in which an action hurts the instigator and benefits the recipient, will emerge provided the perceived stake is sufficiently positive. In contrast, interdependent selfishness, in which an action benefits an instigator and hurts the recipient, will emerge provided the perceived stake is not too positive. Interdependent spite, in which an action hurts both the instigator and recipient, is the most damaging kind of conflict. Such spiteful conflicts emerge when the perceived stake is sufficiently negative. Conflicts may be resolved by shifting perceptions, particularly when they arise from inaccurate perceptions in the first place (e.g. inaccurate knowledge about the stake, *s*, or the downstream effects of an action, *w*_a_ and *w*_r_).

	action has positive effect on instigator, *w*_a_ > 0	action has negative effect on instigator, *w*_a_ < 0
action has positive effect on recipient: *w*_r_ > 0	**Interdependent mutualism.** Perform action if perceived interdependence is not too negative: *s* > −|*w*_a_|/|*w*_r_|	**Interdependent altruism.** Perform action if perceived interdependence is sufficiently positive: *s* > |*w*_a_|/|*w*_r_|
action has negative effect on recipient: *w*_r_ < 0	**Interdependent selfishness.** Perform action if perceived interdependence is not too positive: *s* < |*w*_a_|/|*w*_r_|	**Interdependent spite.** Perform action if perceived interdependence is sufficiently negative: *s* < −|*w*_a_|/|*w*_r_|

More generally, the problem with individual assessment as a tool for resolving conflict is that it is costly, especially when the burden of assessment falls on individuals. Moreover it can be vulnerable to errors—and individuals may even be tempted to strategically encourage misperceptions (for example, by duping a partner about the extent of their shared interests). Shifting the costs of assessment to institutions can mitigate this problem (e.g. [98]), and this approach is especially useful where there is an unlikely but costly risk, as in the example discussed above. Examples include security checkpoints at airports and other locations, checking a taxi driver's ID card, performing a check for undisclosed criminal records when hiring an employee, and so on. Providing people with low-cost actions to improve perceptions in these situations can limit any conflict to cases where it is unavoidable, and prevent group-level conflicts of the type described above. Institutional efforts to reduce uncertainty and improve perceptions are also increasingly a feature of online information ecosystems, including online rating and reputation tools, accuracy assessments of news stories or tools for reporting antisocial behaviour [99–101]. Nonetheless, as the continued problems with online environments illustrate, such tools can be difficult to implement well, and such efforts are counterbalanced by the speed, anonymity and interconnectedness of online interactions, which make interdependence increasingly hard to assess. The development of tools that allow people to reduce their uncertainty and improve the accuracy of their perceptions of interdependence, particularly in online environments, is a key challenge for researchers interested in reducing interdependent conflict.

## Data Availability

This article has no additional data.
